# Trapped Embolic Protection Device: A Salvage Technique

**DOI:** 10.7759/cureus.9228

**Published:** 2020-07-16

**Authors:** Anna Luisa Kuhn, Ajit S Puri, Katyucia De Macedo Rodrigues, Francesco Massari, Jasmeet Singh

**Affiliations:** 1 Department of Neurointerventional Radiology, University of Massachusetts, Worcester, USA; 2 Division of Neuroimaging and Intervention, Department of Radiology, University of Massachusetts, Worcester, USA; 3 Division of Neurointerventional Radiology, University of Massachusetts, Worcester, USA

**Keywords:** carotid stent, carotid arterial disease, carotid stenting, internal carotid artery (ica), carotid angioplasty, carotid calcification, carotid atherosclerosis

## Abstract

After carotid artery stenting, retrieval of the embolic protection device can sometimes be difficult due to incomplete stent expansion, stent fracture, vasospasm, and vessel tortuosity. In this technical report, we describe a novel rescue technique used in a patient with diffuse calcific atherosclerosis of the left common and proximal left internal carotid arteries who underwent left internal carotid artery stenting with cerebral protection and in whom, due to an under-expanded proximal carotid stent strut in relation to a densely calcified plaque, we were initially unable to advance the retrieval device.

## Introduction

Carotid artery stenting is the preferred treatment for patients with symptomatic carotid stenosis and who are considered “high risk” for carotid endarterectomy [1]. The use of embolic protection devices (EPDs) is standard of care in carotid stenting and minimizes the risk for procedure-related strokes [2]. Retrieval of the EPD can sometimes be challenging, especially in the setting of incomplete stent expansion, stent fracture, vasospasm, and vessel tortuosity. Having a rescue plan in such scenarios is crucial.

## Technical report

**Figure 1:**
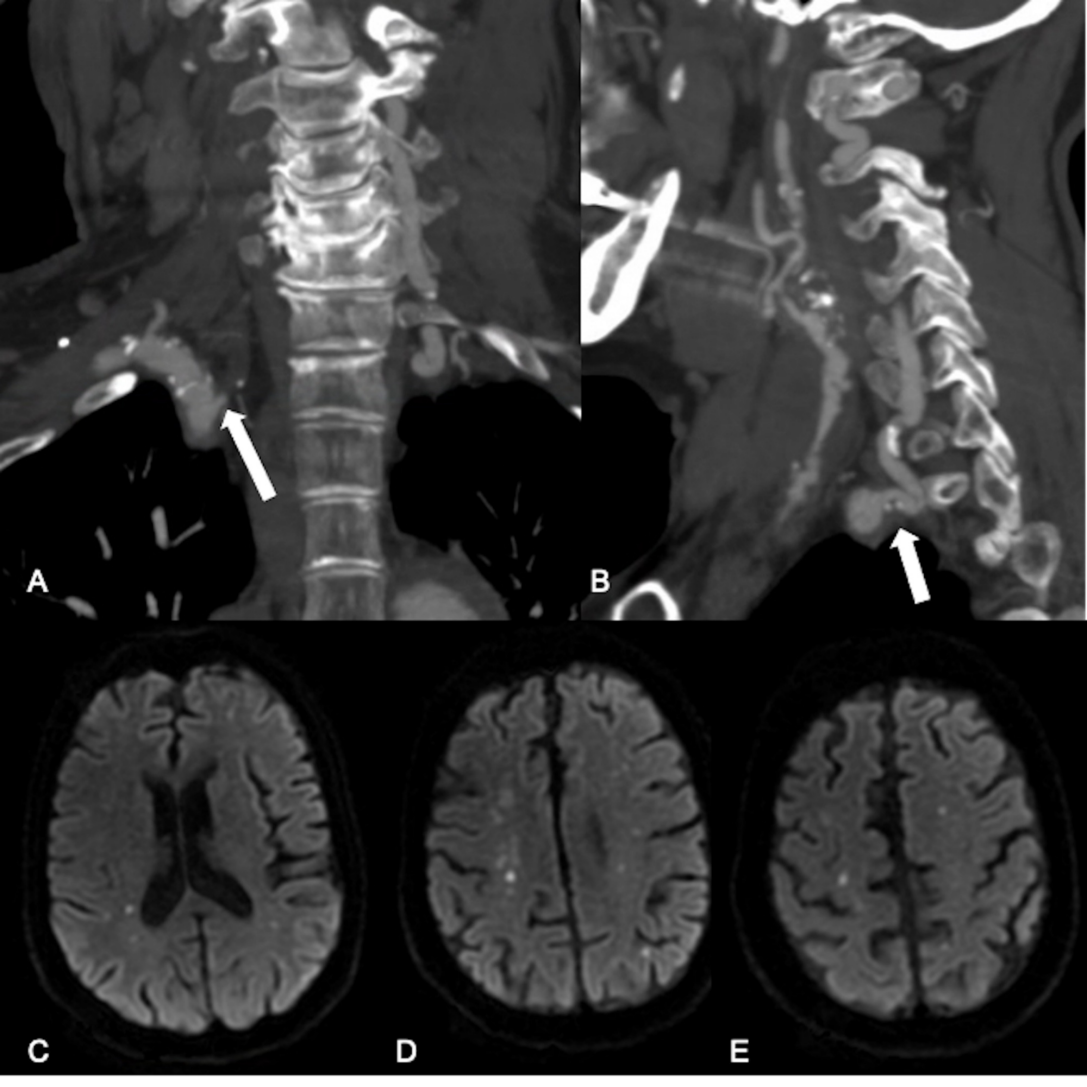
Bilateral vertebral artery disease and bihemispheric anterior circulation infarcts A, B - CT angiography coronal and sagittal reconstructions: occlusion of the right vertebral artery at its origin with visualization of a vessel stump (A, arrow). Moderate stenosis at the origin of the left vertebral artery due to mixed calcified and non-calcified plaque (B, arrow). C, D, E - MRI DWI axial slices: magnetic resonance imaging of the brain reveals multiple small infarcts in both cerebral hemispheres (C-E).

A middle-aged patient presented to our clinic with a past medical history of multiple prior strokes, hypertension, hyperlipidemia, chronic kidney disease, and chronic obstructive pulmonary disease (COPD). The patient developed stroke symptoms while in rehabilitation for a fractured hip after a fall. On work-up, the patient was found to have extensive atherosclerotic changes throughout the cervical vasculature, including bilateral severe to critical internal carotid artery (ICA) stenoses, right vertebral artery occlusion and moderate stenosis of the left vertebral artery origin (Figure 1A and 1B). MRI of the brain revealed multiple small infarcts in both cerebral hemispheres (Figure 1C-E).

**Figure 2:**
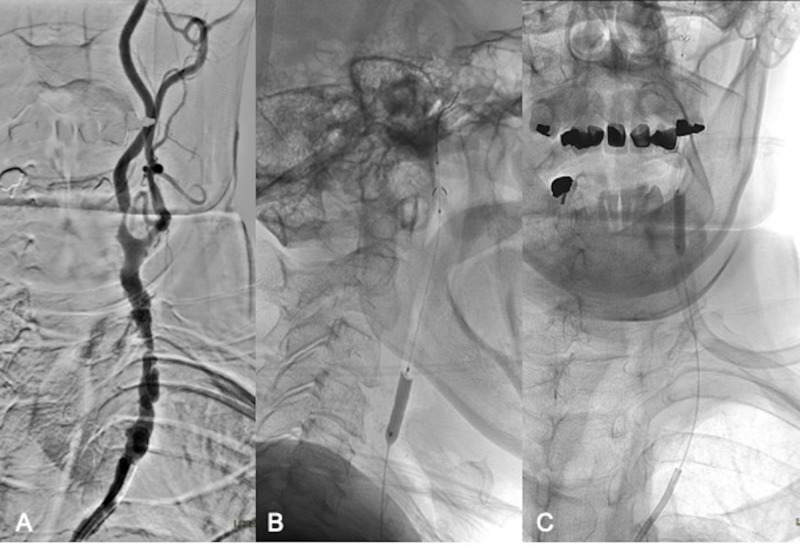
Left CCA and ICA anatomy, EPD placement and pre-stent balloon angioplasty Severe calcific atherosclerotic disease of the left CCA and ICA (A). Successful placement of an embolic protection device in the distal cervical left ICA and pre-stent angioplasty (B and C). CCA - common carotid artery; ICA - internal carotid artery; EPD - embolic protection device

The patient underwent right carotid endarterectomy but unfortunately developed hoarseness postoperatively due to vocal cord palsy. The patient also had a preexisting type two aortic dissection. For the treatment of her high-grade left internal carotid artery (ICA) stenosis, she opted for minimally invasive endovascular stenting. The procedure was performed under monitored anesthesia care (MAC) using dexmedetomidine (Precedex™) with continuous cardiovascular monitoring. A 65 cm long 6 French Super Arrow-Flex® sheath (Teleflex®, Morrisville, NC) was placed in the proximal descending aorta and a 6 French Envoy® guiding catheter (DePuy Synthes, Raynham, MA) was used to select the origin of the left common carotid artery (CCA). There was a severe diffuse atherosclerotic disease of the left CCA and ICA with extensive irregular atherosclerotic plaque formation and multiple plaque ulcers (Figure 2A). Two areas of critical, more than 90% stenosis, were noted in the proximal left CCA and left ICA origin. The Envoy catheter was successfully advanced beyond the origin of the left CCA. Through this catheter, a 5 mm Emboshield NAV™ embolic protection system (Abbott Vascular, Santa Clara, CA) was carefully navigated into the high cervical left ICA and deployed uneventfully. A 4.5 mm x 20 mm Monorail™ over-the-wire balloon (Boston Scientific, Natick, MA) was advanced over the EPD wire to perform pre-stenting angioplasty of the CCA and proximal left cervical ICA (Figure 2B and 2C).

**Figure 3:**
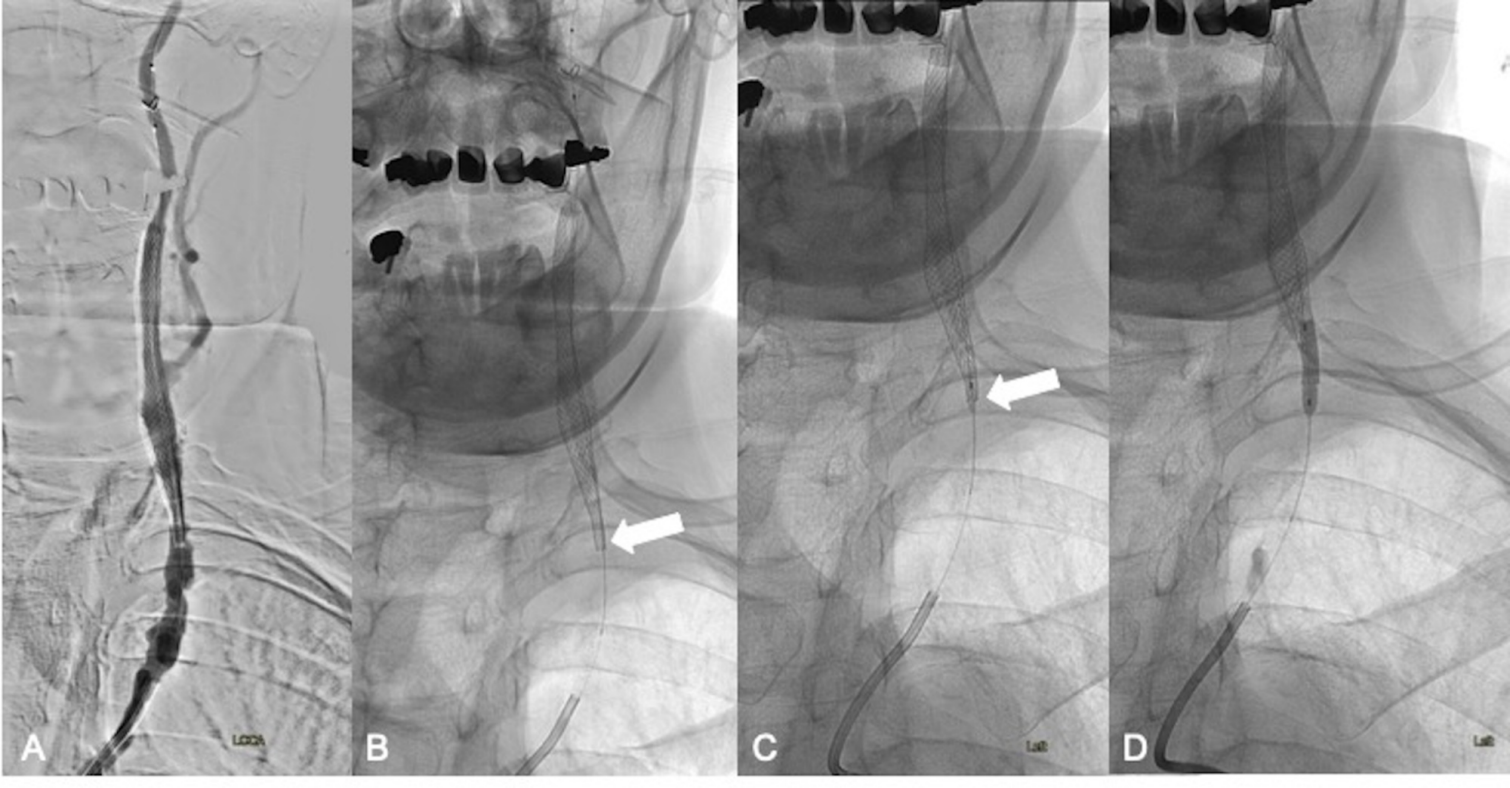
Left ICA stenting and post-stent balloon angioplasty Placement of an 8 x 36 mm Carotid Wallstent (Boston Scientific) spanning from the C3 to C7 level and extending into the left CCA (A). Incomplete opening of the proximal stent struts (B, arrow) and serial angioplasty of the proximal stent using a 1.5 mm x 9 mm (C, arrow) and 3 mm x 15 mm Gateway over-the-wire balloons (Stryker Neurovascular) (D). ICA - internal carotid artery; CCA - common carotid artery

An 8 x 36 mm Carotid Wallstent® (Boston Scientific) was then deployed in the left cervical ICA, spanning from the C3 to C7 level and extending into the left CCA (Figure 3A). Due to the previously described atherosclerotic plaque in the CCA, it was noted that the proximal portion of the stent did not appropriately open (Figure 3B, arrow). Attempts to advance the 4.5 mm x 20 mm Monorail over-the-wire balloon (Boston Scientific) to perform angioplasty at the level of the proximal stent and open the metal struts were unsuccessful. The balloon was removed, and a docking Traxcess® wire (Traxcess 14, Microvention® Terumo, Tustin, CA) was connected to the end of the monorail wire from the EPD. Serial angioplasty of the stent was then performed at the proximal opening of the stent using 1.5 mm x 9 mm (Figure 3C, arrow) and 3 mm x 15 mm Gateway over-the-wire balloons (Stryker® Neurovascular, Kalamazoo, MI) (Figure 3D).

**Figure 4:**
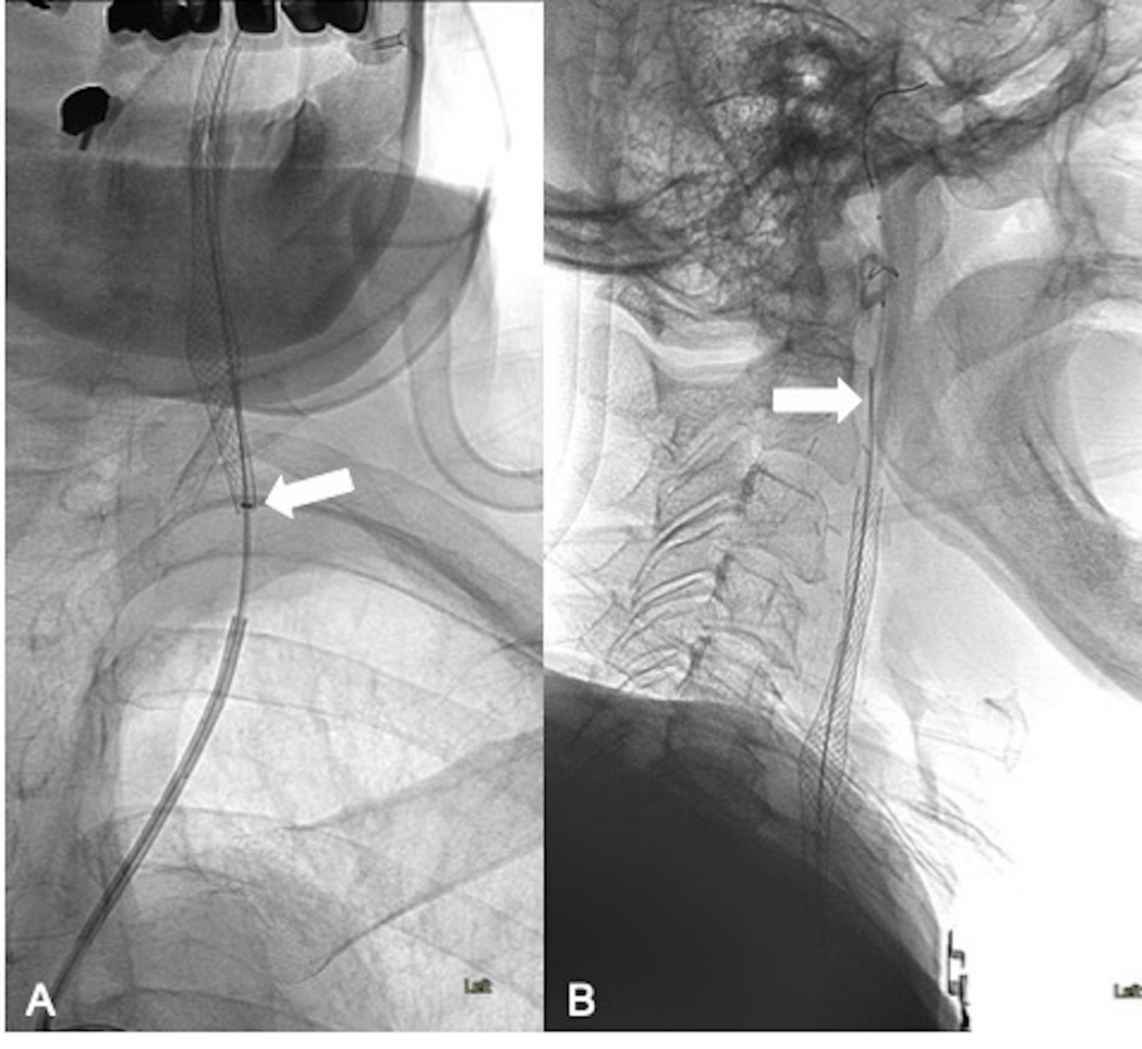
Capture of the trapped EPD As the capture device was still not able to enter the stent, a soft-tipped 5 French Sofia intermediate/distal access catheter (MicroVention Terumo) was advanced into the stent (A, arrow) using "buddy-wire" technique with a 0.035-inch Terumo Glidewire (Terumo Medical, Somerset, NJ) by the side of the embolic protection device wire (B, arrow). EPD - embolic protection device

Despite successful angioplasty, the capture device for the EPD would not advance over the wire. We then advanced a 125 cm soft-tipped 5 French Sofia® intermediate/distal access catheter (MicroVention® Terumo) (Figure 4A, arrow) and used buddy wire technique with a 0.035-inch Terumo Glidewire® (Terumo Medical, Somerset, NJ) by the side of the embolic protection device wire (Figure 4B, arrow) to pass through the stent and successfully capture the embolic protection device. Once the EPD was removed, the deployed stent was re-accessed with a microwire. 

**Figure 5:**
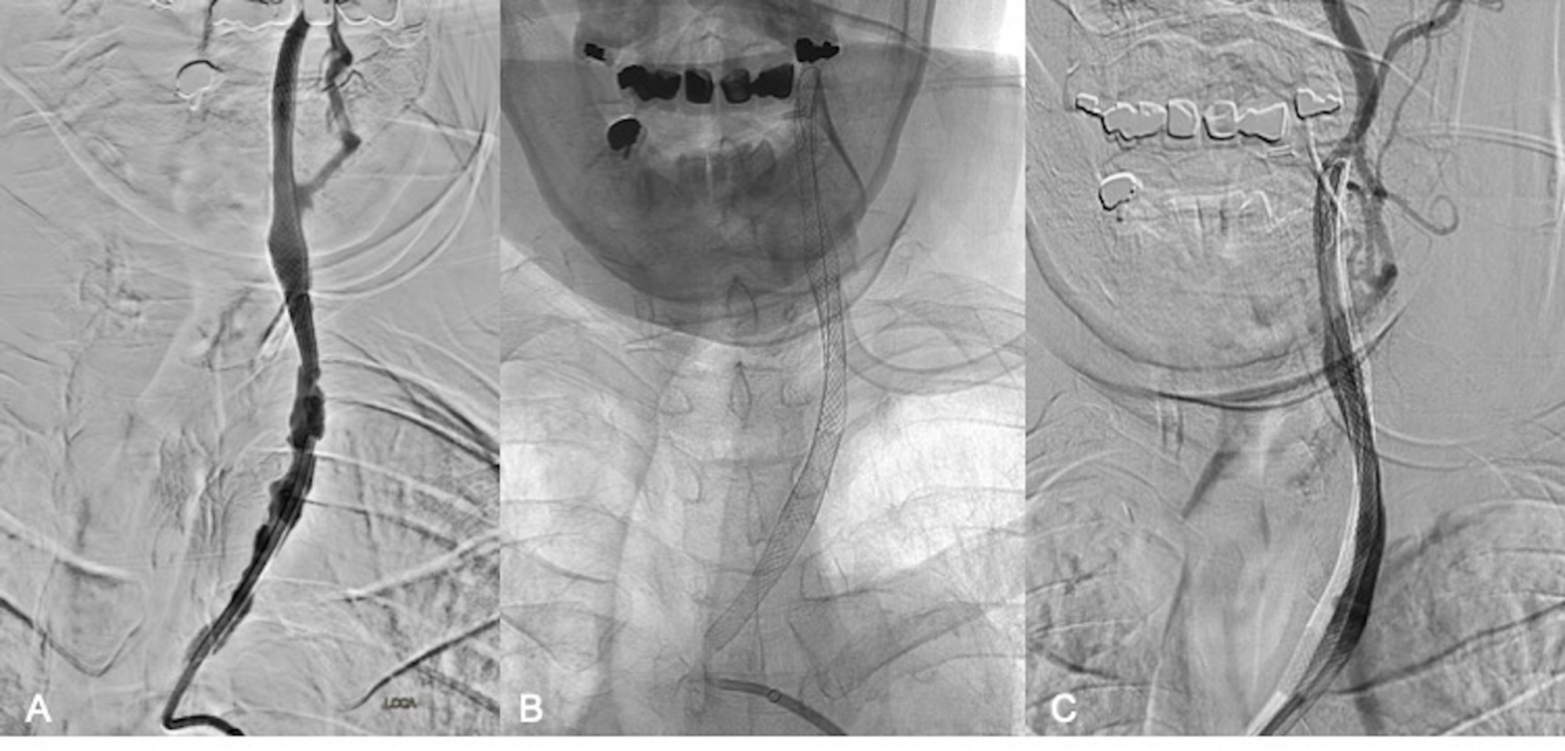
Left CCA stenting After the recapture of the embolic protection device, placement of two additional overlapping 8 x 29 mm Carotid Wallstents (Boston Scientific) to cover the diseased left CCA (A-C). The final angiogram upon completion of the procedure is shown in image C. CCA - common carotid artery

Two 8 x 29 mm overlapping Carotid Wallstents (Boston Scientific) were then placed across the severely diseased left CCA (Figure 5A-C) up to its origin in the aorta and angioplasty of the stented vessel segment was performed using a 5 mm x 20 mm Monorail balloon (Boston Scientific). The patient tolerated the procedure well and remained neurologically intact. After one night of in-hospital observation, the patient was discharged home the next day on a dual antiplatelet regimen consisting of ASA 81 mg and Plavix® 75 mg daily.

## Discussion

There have been descriptions of rescue strategies to retrieve trapped embolic protection filters including external carotid compression, rotation of the head to the contralateral side to change carotid angulation, use of different catheters like the vertebral and JR-four catheters and carotid endarterectomy (CEA) [3-8]. CEA for EPD retrieval can be performed safely in an emergent setting [7, 8]. However, rescue strategies are mainly described in case reports and, therefore, the optimal management of a retained EPD remains a challenge. Calcified plaques and vessel tortuosity are independent risk factors associated with the difficult retrieval of embolic protection devices [9]. Both of these factors were present in our case. Also, since our guiding catheter position was very proximal, we anticipated some difficulty during device retrieval and, therefore, had a backup action plan in mind before initiating the procedure. Dauherty et al. encountered a similar case scenario in which they used a 5 French 125 cm vertebral catheter to recapture and remove a trapped Emboshield™ embolic protection device (Cordis Endovascular, Hialeah, FL) [4]. They had to advance the 125 cm 5 French vertebral catheter over the 180 cm Emboshield wire. Because only approximately 60 cm of the 180 cm wire was exposed outside the guiding sheath, they were unable to pin or hold the wire as the diagnostic catheter was advanced. Our technique offers more control as the attachment of the Traxcess docking wire (Microvention Terumo) essentially changes a Monorail system into an exchange microwire system. Use of this system allowed us to utilize the smaller over-the-wire Gateway balloon for angioplasty of the stent struts. This ultimately allowed us to pass the 5 French Sofia intermediate/distal access catheter and capture the EPD.

## Conclusions

Having a "plan B" can make all the difference between a successful and unsuccessful procedural outcome. The navigability of a distal access catheter makes it a useful tool not only for intracranial interventions but also as a rescue tool to recapture a trapped EPD. Our rescue technique has not been described in the literature and could prove very useful in difficult situations during carotid artery stenting. Other helpful considerations include the use of docking wires which provide extra length to EPD wires and the "buddy-wire" technique which can help provide stability and guidance for a catheter.
